# Perceptions Regarding Proposed Changes to ACGME Program Requirements for Emergency Medicine

**DOI:** 10.51894/001c.164058

**Published:** 2026-08-01

**Authors:** Cheri Lotfi, Chikezie Onyenso, Zechariah Jean, Charles Adams, Zachary Jarou, Hrag Churukian, Gino Etta

**Affiliations:** 1 Henry Ford Warren Hospital

## 29

### Background

The Accreditation Council for Graduate Medical Education (ACGME) Review Committee for Emergency Medicine has proposed substantial revisions to residency requirements, including extending training to 48 months, mandating rotations in both high- and low-resource emergency departments, expanding procedural minimums, and requiring scholarly dissemination. Motivations include declining American Board of Emergency Medicine (ABEM) pass rates, shorter clinical shifts and a need for broader preparedness. The impact of these changes on residents, applicants, and faculty remains largely uncertain.

### Objective

To assess the perceptions of EM-bound medical students, residents, and faculty regarding the proposed 2025 ACGME Emergency Medicine (EM) program requirement changes.

### Methods

A cross-sectional survey was distributed nationally via local program outreach and the Emergency Medicine Residents’ Association (EMRA). Respondents included EM-bound medical students, residents, and faculty. Attitudes were measured using a 5-point Likert scale across domains of training length, procedural minimums, site diversity, scholarly activity, and wellbeing. Descriptive statistics and chi-square tests were used to compare groups.

### Results

Ninety respondents completed the survey (nmedical students = 7, nPGY-1 = 29, nPGY-2 = 9, nPGY-3 = 17, nfaculty = 28). Support for extending training to 48 months was low (overall mean 2.3 ± 1.3), with trainees less supportive than faculty (2.1 vs. 2.9, *p* = 0.002). Requirements for rotations in high/low-resource EDs were rated favorably (3.9 ± 0.8), particularly by trainees. Procedural minimums such as arterial lines (3.7 ± 1.1, *p* = 0.01) and intraosseous access (3.4 ± 1.1, *p* = 0.04) were supported, while neonatal resuscitation received lower ratings (2.9 ± 1.2). The scholarly project requirement was the least supported change (2.2 ± 1.0). Most respondents believed the revisions would negatively affect resident wellbeing (3.7 ± 1.1; trainees 4.0 vs. faculty 3.0, *p* < 0.001). Overall, the proposal received a low rating (2.6 ± 1.2), with trainees less favorable than faculty (2.4 vs. 3.0, *p* = 0.01).

### Conclusions

Stakeholders expressed mixed perceptions of proposed ACGME changes. Although expanded procedural exposure and clinical site diversity were viewed favorably, substantial concerns regarding training duration, scholarly activity requirements, and resident wellbeing, especially among trainees, highlight the need for careful consideration.

